# Macroscopic Kinetics of Pentameric Ligand Gated Ion Channels: Comparisons between Two Prokaryotic Channels and One Eukaryotic Channel

**DOI:** 10.1371/journal.pone.0080322

**Published:** 2013-11-19

**Authors:** Kurt T. Laha, Borna Ghosh, Cynthia Czajkowski

**Affiliations:** 1 Department of Anesthesiology, University of Wisconsin, Madison, Wisconsin, United States of America; 2 Department of Neuroscience, University of Wisconsin, Madison, Wisconsin, United States of America; 3 Biophysics Training Program, University of Wisconsin, Madison, Wisconsin, United States of America; University of Waterloo, Canada

## Abstract

Electrochemical signaling in the brain depends on pentameric ligand-gated ion channels (pLGICs). Recently, crystal structures of prokaryotic pLGIC homologues from *Erwinia chrysanthemi* (ELIC) and *Gloeobacter violaceus* (GLIC) in presumed closed and open channel states have been solved, which provide insight into the structural mechanisms underlying channel activation. Although structural studies involving both ELIC and GLIC have become numerous, thorough functional characterizations of these channels are still needed to establish a reliable foundation for comparing kinetic properties. Here, we examined the kinetics of ELIC and GLIC current activation, desensitization, and deactivation and compared them to the GABA_A_ receptor, a prototypic eukaryotic pLGIC. Outside-out patch-clamp recordings were performed with HEK-293T cells expressing ELIC, GLIC, or α_1_β_2_γ_2L_ GABA_A_ receptors, and ultra-fast ligand application was used. In response to saturating agonist concentrations, we found both ELIC and GLIC current activation were two to three orders of magnitude slower than GABA_A_ receptor current activation. The prokaryotic channels also had slower current desensitization on a timescale of seconds. ELIC and GLIC current deactivation following 25 s pulses of agonist (cysteamine and pH 4.0 buffer, respectively) were relatively fast with time constants of 24.9±5.1 ms and 1.2±0.2 ms, respectively. Surprisingly, ELIC currents evoked by GABA activated very slowly with a time constant of 1.3±0.3 s and deactivated even slower with a time constant of 4.6±1.2 s. We conclude that the prokaryotic pLGICs undergo similar agonist-mediated gating transitions to open and desensitized states as eukaryotic pLGICs, supporting their use as experimental models. Their uncharacteristic slow activation, slow desensitization and rapid deactivation time courses are likely due to differences in specific structural elements, whose future identification may help uncover mechanisms underlying pLGIC gating transitions.

## Introduction

Pentameric ligand gated ion channels (pLGICs) mediate excitatory and inhibitory synaptic transmission and their evolutionary precursors have been identified in several bacteria [1]. These channels, comprised of five homologous subunits arranged pseudo-symmetrically around a central ion conducting pore, are structurally adapted to rapidly convert chemical signals (i.e. the binding of ligands) into electrical signals (i.e. ion flow through a central pore). The eukaryotic pLGICs are commonly referred to as Cys-loop receptors and include cation-selective ion channels (nicotinic acetylcholine (nACh) receptors and serotonin-type 3A (5-HT3A) receptors) and anion-selective channels (γ-aminobutyric acid-type A receptors (GABA_A_Rs), glycine receptors, and an invertebrate channel, GluCl). Two prokaryotic pLGIC homologues have been identified, ELIC and GLIC, from the plant pathogen *Erwinia chrysanthemi* and the cyanobacterium *Gloeobacter violaceus*, respectively [1], [2].

Since the first cloning of a Cys-loop receptor in 1983 [3], researchers have sought to understand how their structure dictates their intricate function. The prokaryotic channels, which can be purified in large amounts, provided the first high-resolution X-ray crystal structures of a full-length pLGIC. ELIC has been crystallized in a presumed closed state [4] and GLIC in an apparent open state [5], [6]. Comparison of the structures reveals distinct conformational changes in the extracellular-binding domain and transmembrane domain that have been used to predict closed to open pLGIC gating mechanisms.

Before structural insights obtained from these prokaryotic homologues can be fully extrapolated to eukaryotic pLGICs, it is important that we establish how well the functional properties of prokaryotic channels mimic the properties of eukaryotic channels. ELIC and GLIC are cationic channels opened by primary amines and protons, respectively [2], [7]. Both channels are blocked by several of the same inhibitors as the eukaryotic pLGICs [8], [9]. Additionally, GLIC is modulated by anesthetics and alcohols [10], [11] and ELIC by benzodiazepines [12].

Upon agonist binding, eukaryotic channels make rapid transitions from closed to open states, and following prolonged agonist exposure accumulate into desensitized states where ligand is bound but the channel is closed. Although the kinetics and dominant conformational states of eukaryotic pLGICs have been examined in detail, functional characterization of ELIC and GLIC is limited. Only a handful of investigations aimed at measuring the time course of ELIC or GLIC current responses have been reported [2], [7], [13], [14], [15] and results from these studies differ, especially for GLIC. In addition, no study to date has directly compared eukaryotic and prokaryotic pLGICs in the same expression system using ultra-fast ligand exchange.

In order to establish a reliable foundation for comparing the functional properties between these receptors, we measured the kinetics of current activation, desensitization, and deactivation for ELIC, GLIC and GABA_A_Rs expressed in the mammalian cell line HEK-293T, a common platform for patch-clamp recordings. A comparison between these prokaryotic channels and GABA_A_R is useful because GABA_A_R exhibits channel kinetics that are representative of most eukaryotic Cys-loop receptors and the endogenous GABA_A_R agonist, GABA, is capable of activating ELIC as well [7]. Here, we show the prokaryotic pLGICs undergo agonist-mediated gating transitions to open and desensitized states similar to GABA_A_Rs but with slower current onset kinetics.

## Methods

### Expression in *Xenopus laevis* and Two-Electrode Voltage Clamp Recording

Rat cDNAs encoding GABA_A_R subunits α1, β2, and γ2L were subcloned in pUNIV vector [16]. ELIC cDNA in pET26b was kindly provided by Dr. Raimund Dutzler, University of Zurich. The DNA sequence encoding GLIC (residues 44–359) was extracted by PCR amplification from *G. violaceus* cells (ATCC, Manassas, VA). ELIC and GLIC DNA sequences were subcloned into the pUNIV vector and were preceded by the DNA sequence encoding the signal peptide of the GABA_A_R β2 subunit.

Capped cRNAs from NotI digested ELIC, GLIC and GABA_A_R α1, β2, γ2L subunits were transcribed in vitro using the mMessage mMachine T7 kit (Life Technologies (Ambion), Carlsbad, CA). *Xenopus laevis* oocytes were injected 24 hours after harvest with 27 nl of cRNA. ELIC or GLIC cRNA was injected at a concentration of 50 ng/µl. For GABA_A_Rs, an injection cocktail was prepared by combining α1, β2 and γ2L subunits in 1∶1∶10 ratio with the final concentration of α1 and β2 subunits being 2 ng/µl and γ2L being 20 ng/µl. Injected oocytes were incubated at 16° C in ND96 (5 mM HEPES pH 7.4, 96 mM NaCl, 2 mM KCl, 1 mM MgCl_2_, 1.8 mM CaCl_2_) supplemented with 100 µg/ml of gentamycin and 100 µg/ml of bovine serum albumin for 2–5 days before use for electrophysiological recordings.

For two-electrode voltage clamp recordings, oocytes were perfused continuously with ND96 at pH 7.4–7.6 at a flow rate of 5 ml/min in a bath volume of 200 µl. Oocytes expressing GABA_A_Rs were voltage clamped at −80 mV and those expressing ELIC and GLIC were clamped at −60 mV. Borosilicate glass electrodes (Warner Instruments, Hamden, CT) used for recordings were filled with 3 M KCl and had resistances of 0.4 to 1.0 MΩ. Electrophysiological data were collected using GeneClamp 500 (Axon Instruments, Foster City, CA) interfaced to a computer with a Digidata 1200 A/D device (Axon Instruments), and were recorded using the Whole Cell Program, version 4.0.2 (provided by J. Dempster, University of Strathclyde, Glasgow, UK). Oocytes were first stabilized by repeated pulses of a low ligand concentration (1 µM GABA for GABA_A_Rs, 100 µM cysteamine for ELIC, and pH 5.5 for GLIC) until currents varied by less than 10%.

GABA and cysteamine concentration-response curves were determined by measuring currents elicited by application of 5–7 concentrations of GABA or cysteamine separated by 3–7 min washes to oocytes expressing GABA_A_Rs and ELIC respectively. Each current response was scaled to a low, non-desensitizing concentration of GABA or cysteamine applied just before the test concentration to correct for any drift in responsiveness over the course of the experiment. Concentration-response data were fit by the following equation: I = I_max_/[1+(EC_50_/[A]^n^_H_)], in which I is the peak response to a given drug concentration, I_max_ is the maximal amplitude of current, EC_50_ is the drug concentration that produces the half-maximal response, [A] is drug concentration, and n_H_ is the Hill coefficient. Proton induced currents from GLIC expressing oocytes were measured by perfusing ND96 buffered at pH 6.5–3.8. For pH 5.0–3.8 HEPES was replaced with 5 mM Na Citrate as the buffering agent. For pH 6.5–6.0 5 mM MES was used as the buffering agent. pH–response curves were obtained by successive applications of 5–6 different pH pulses, separated by 3–7 min washes and fit to the equation I = I_max_/[1+10^∧^((pH-pH_50_)×n_H_)]

where I is the peak response at a given pH, I_max_ is the maximum amplitude of current, pH_50_ is the pH inducing half maximal response, and n_H_ is the Hill coefficient. GraphPad Prism 4 (GraphPad Software Inc., San Diego, CA) was used for data analysis and curve fitting.

### Cell Culture and Transfection of HEK-293T cells

Human embryonic kidney (HEK-293T) cells were cultured in Minimum Essential Medium Eagle with Earle's salts (Mediatech, Manassas, VA) supplemented with 10% fetal bovine serum (Atlanta Biologics, Atlanta, GA) and Penicillin-Streptomycin-Glutamine (Sigma-Aldrich, St. Louis, MO) in a 37°C incubator under a 5% CO_2_ atmosphere. Cells were plated onto 35 mm dishes coated with poly-L-lysine. 18–24 hours later they were transfected using Lipofectamine 2000 (Invitrogen, Carlsbad, CA) with 1 µg of ELIC or GLIC cDNA (in the pUNIV vector). For expression of the eukaryotic channel rat α1β2γ2L GABA_A_R, the following amounts of cDNA were transfected: 1 µg of α1, 1 µg β2, and 3 µg of γ2L. In all cases 250 ng eGFP was included as a marker of transfection. Cells were recorded from 24–72 hours post-transfection.

### Patch-clamp recording from HEK-293T cells

All recordings from HEK-293T cells were collected from excised outside-out patches held at −60 mV. Recordings were made using borosilicate glass pipettes filled with (in mM): 140 KCl, 10 EGTA, 2 MgATP, 20 phosphocreatine and 10 HEPES, pH 7.4. For recordings with GABA_A_R, the perfusion solution contained (in mM) 145 NaCl, 2.5 KCl, 2 CaCl_2_, 1 MgCl_2_, 10 HEPES and 4 Glucose, pH 7.4. The bath solution used during recordings of ELIC and GLIC consisted of (in mM) 140 NaCl, 2.8 KCl, 2 CaCl_2_, 2 MgCl_2_, 10 glucose, and 10 HEPES and was buffered at pH 7.6. For ELIC, the bath solution contained 2 mM BaCl_2_, in place of CaCl_2_, because Ca^2+^ is a potent modulator of ELIC currents compared to Ba^2+^ [17]. For the activation of ELIC, cysteamine was dissolved in the bath solution and 10 mM DTT was added to prevent oxidation of its sulfhydryl group. For the activation of GLIC a solution buffered with sodium citrate between pH 5.0 and pH 4.0 instead of HEPES was utilized. Rapid solution exchange was accomplished by using a multi-barreled flowpipe array (Vitrodynamics, Rockaway, NJ) mounted on a piezoelectric bimorph (Vernitron, Bedford, OH). A computer-controlled constant current source drove the bimorph to move solution interfaces over the patch with 10–90% exchange times of 300 µs, as measured by the liquid junction current at the open pipette tip. At least 20 s washes were applied between applications of agonists. Currents were low-pass-filtered at 5 kHz with a four-pole Bessel filter, and data were collected at 20 kHz using an Axopatch 200B amplifier (Axon Instruments) and a Digidata 1322A (Axon Instruments) controlled by Axograph X software (Axograph, Sydney, Australia). Displayed traces are ensemble averages of several (3 to 15) sweeps.

### Analysis of macroscopic kinetics-curve fitting

The ensemble average of current responses was taken for a given patch and was fit using Axograph X. Current activation was fit from the onset of current until the current reached its maximum amplitude. For GLIC and GABA_A_R current activation was best fit with a bi-exponential equation (Y = A_1_×(1− e^−t/τ1^)+A_2_×(1−e^−t/τ2^)), where t is time, Y is the total current amplitude at a given time, τ1 is the time constant of the fast component of rise, A1 is the relative amplitude of the fast component, τ2 is the time constant of the slow component of rise, A2 is the relative amplitude of the slow component. The current activation for ELIC was best fit with a single exponential equation (Y = A×(1− e^−t/τ^)). For analysis of current desensitization the time of onset of desensitization was set to zero, and the time course of desensitization was fit with a single exponential equation (Y = A_1_×e^−t/τ1^+C) or bi-exponential equation (Y = A_1_×e^−t/τ1^+A_2_×e^−t/τ2^+C), where C is a constant that accounts for the amplitude of current that remains. For analysis of current deactivation the time of agonist removal was set to zero. The time course of deactivation for GLIC and GABA_A_R was fit with a bi-exponential equation (Y = A_1_×e^−t/τ1^+A_2_×e^−t/τ2^), whereas ELIC current deactivation was best fit with a single exponential equation (Y = A_1_×e^−t/τ1^). A weighted time constant (τ_w_) was also calculated for each analysis, (τ_w_ = (A_1_/(A_1_+A_2_))×τ_1_+(A_2_/(A_1_+A_2_))×τ_2_). This value was used for making direct comparisons of the overall time course of the various phases of current development.

### Statistics

Graphpad Prism 4 was employed for performing statistical significance tests. Student's t-tests (unpaired, two-tailed) were performed with macroscopic deactivation times from each channel following different lengths of exposure to agonist. All values are presented as the mean ± SEM.

## Results

**Figure 1:**
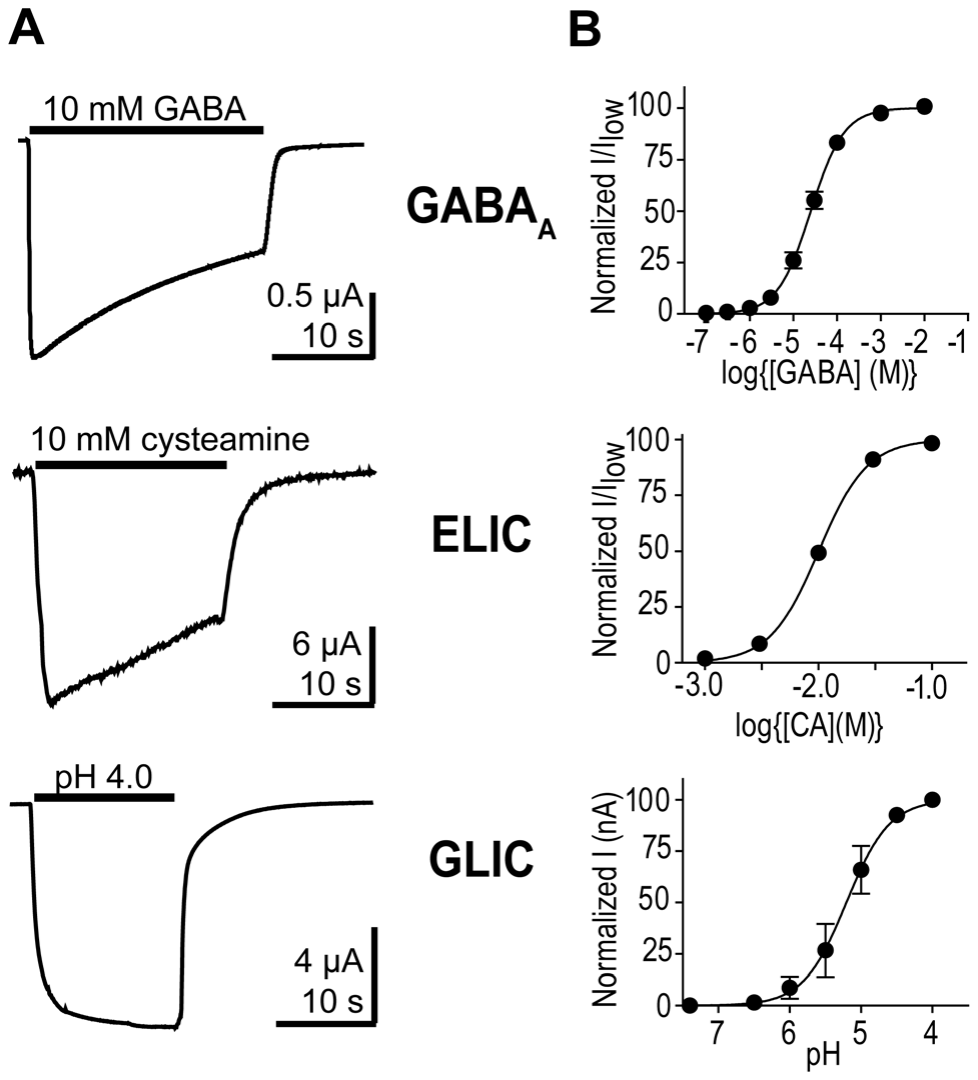
GABA_A_ receptor, ELIC, and GLIC current responses measured using two-electrode voltage clamp. A) Representative currents from the GABA_A_ receptor, ELIC, and GLIC expressed in oocytes evoked by saturating concentrations of agonist (black bar). B) Concentration response curves for the GABA_A_ receptor, ELIC, and GLIC. CA = cysteamine.

Initially, we examined currents evoked by GABA, cysteamine, and changes in pH from the GABA_A_R, ELIC, and GLIC, respectively, expressed in *X. laevis* oocytes (Figure 1A). The agonist EC_50_ values for each channel were determined from concentration response curves (25±4 µM GABA; 10±0.1 mM cysteamine; pH 5.1±0.05) (Figure 1B). Because of slow solution exchange when recording from oocytes using two-electrode voltage clamp, differences in current time courses are difficult to distinguish and interpret. Thus, we turned to outside-out patch-clamp recordings from HEK-293T cells and ultra-fast solution exchange, which allows precise measurement of current macroscopic kinetics.

### GABA macroscopic kinetics

**Figure 2:**
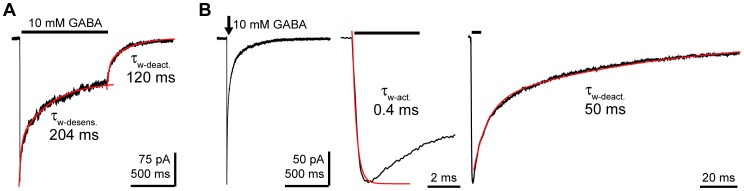
Outside-out patch-clamp recordings from HEK-293T cells expressing GABA_A_Rs. A) Current evoked by a 1 second pulse of 10 mM GABA. The current decrease in the presence of agonist (desensitization) and upon agonist washout (deactivation) were fit with bi-exponential functions (red curves) and the weighted time constants for the example trace are shown. B) Current evoked by a 5 millisecond application of 10 mM GABA (black arrow). To the right, expanded traces highlight the current increase during GABA application (activation) and the current deactivation following washout of GABA. The time course of activation was determined by fitting the rising phase with a bi-exponential function (red curve) and the timecoure of deactivation was determined by fitting the current decay after agonist removal with a bi-exponential function (red curve). The weighted time constants for the example traces are shown. The traces shown are the ensemble averages of multiple GABA-evoked currents from a single patch.

When 10 mM GABA is applied to a patch for 1 s, the macroscopic kinetics of the GABA_A_R current has three distinct phases: a fast rising current activation phase with a time constant (τ-activation) of 0.8 ms±0.1 ms, n = 19, a desensitization phase, where current decreases in the presence of agonist, with a weighted time constant (τ_w_-desensitization) of 162±37 ms, n = 10, and a deactivation phase, where current decays back to baseline after removal of GABA, with a weighted time constant (τ_w_-deactivation) of 104±8 ms, n = 10 (Figure 2A). Using a very brief 5 ms pulse of 10 mM GABA, little desensitization occurs and the deactivation phase exhibits a τ-deactivation of 64±5 ms, n = 9 (Figure 2B).

### ELIC macroscopic kinetics

**Figure 3:**
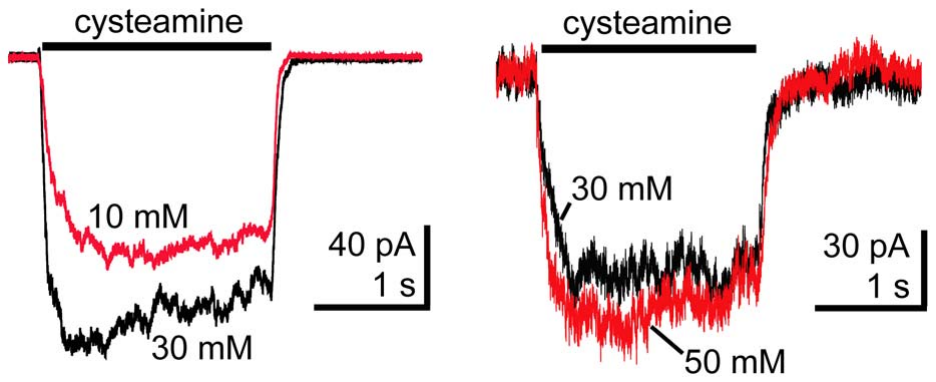
Cysteamine activation of ELIC. Outside-out patch-clamp recordings from HEK-293T cells expressing ELIC. ELIC currents evoked by alternating pulses of 30 mM and 10 mM or 50 mM cysteamine (black bar) are overlaid. Current amplitudes and rise-times evoked by 30 mM and 50 mM cysteamine were similar suggesting 30 mM was saturating.

We examined ELIC currents from outside-out patches evoked by cysteamine using concentrations up to 50 mM (Figure 3). Cysteamine is the most potent agonist that has been identified for ELIC [7]. Patch stability was compromised above 30 mM cysteamine, making it difficult to obtain recordings using higher concentrations. The amplitudes and rise times of the evoked responses were similar when using concentrations of 30 mM or 50 mM cysteamine suggesting 30 mM was saturating. Therefore, we used 30 mM cysteamine in all of our subsequent experiments.

**Figure 4:**
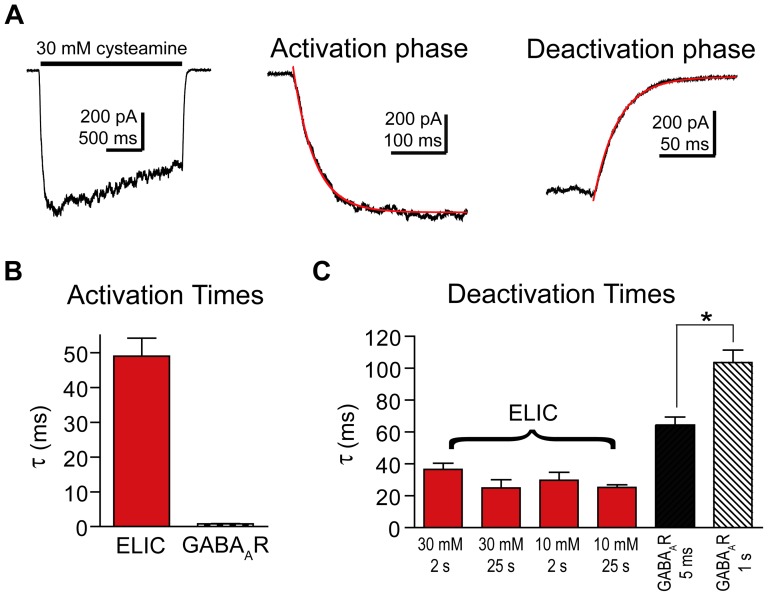
ELIC current activation was slow and deactivation fast compared to GABA_A_Rs. A) Macroscopic ELIC current evoked by a 2 s application of 30 mM cysteamine (black bar) (left). Expanded views depict current activation (center) and deactivation (right) fit with single exponential functions (red). B) The τ-activations for ELIC currents (30 mM cysteamine) and GABA_A_R currents (10 mM GABA) are plotted. Data are mean ± SEM (ELIC: n = 9, GABA_A_R: n = 19). C) The τ-deactivations for ELIC currents evoked by short (2s) and long (25s) pulses of 30 mM and 10 mM cysteamine are plotted. The τ_w_-deactivations for GABA_A_R currents evoked by 5 ms or 1 s applications of 10 mM GABA are also plotted. GABA_A_R current deactivation after a 1 s pulse is significantly slower than after a 5 ms pulse (* p<0.001). Data are mean ± SEM (ELIC 30 mM 2 s: n = 9, ELIC 30 mM 25 s: n = 3, ELIC 10 mM 2 s: n = 6, ELIC 10 mM 25 s: n = 4, GABA_A_R 5 ms: n = 9, GABA_A_R 1 s: n = 10).

Cysteamine activation of ELIC current was fit with a single exponential (Figure 4A). The time course of ELIC activation had a τ-activation of 48±4 ms, n = 9 (Figure 4B), which is several orders of magnitude slower than GABA activation of the GABA_A_R. We measured ELIC current deactivation following a 2 s application (Figure 4A) or a 25 s application of 30 mM cysteamine, which caused the current to desensitize about 2% or 75%, respectively, from the peak amplitude (Figure 4C). For both the 2 s and 25 s applications, ELIC current deactivation was fit with a single exponential and there were no significant differences in their decay (Figure 4C; 30 mM cysteamine 2 s: τ-deactivation = 35±5 ms, n = 9; 25 s: τ-deactivation = 25±5 ms, n = 3). Also, there was no difference in current deactivation when using a lower concentration of cysteamine (10 mM cysteamine 2 s: τ-deactivation = 28±2 ms, n = 6; 25 s: τ-deactivation = 24±3 ms, n = 4). Previous studies on eukaryotic receptors have shown that increasing agonist concentration and duration of application slow the time course of current deactivation due to an increase in the number of channels in the desensitized state [18]. As mentioned earlier, we measured GABA_A_R current deactivation following a 5 ms and a 1 s pulse of 10 mM GABA, which desensitized the currents 9% and 75%, respectively. As expected, GABA current deactivation was significantly slower after longer agonist exposures (64 ms compared to 104 ms; Figure 2 and Figure 4C).

**Figure 5:**
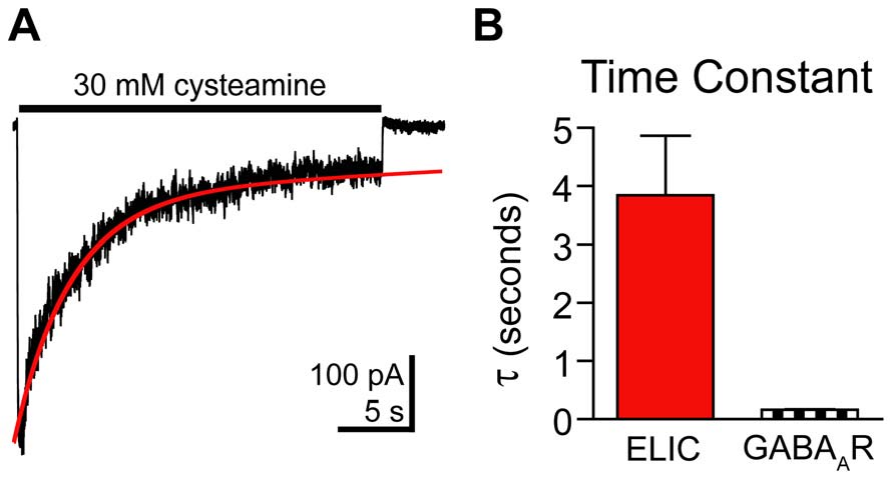
Macroscopic ELIC current desensitization. A) The desensitization phase of ELIC current evoked by a 25 s pulse of 30 mM cysteamine was fit with a single-exponential function (red curve). B) The τ-desensitization for ELIC currents as measured in (A) and the τ_w_-desensitization for GABA_A_R currents measured during a 1s application of 10 mM GABA are plotted. Data are mean ± SEM (ELIC: n = 4, GABA_A_R: n = 10).

During prolonged exposure to 30 mM cysteamine (25 s), the ELIC currents desensitized (Figure 5A and B). The current decay during prolonged agonist exposure was fit with a single exponential with a τ-desensitization of 3.8±1.0 s, n = 4, and 26±9% of the current remained after 25 s. ELIC current desensitization was slower than GABA_A_R current desensitization (Figure 5B). GABA_A_R currents in the presence of 10 mM GABA exhibited a bi-exponential decay with a τ_w_-desensitization of 162±37 ms with about 25% of the current remaining after 1 s.

**Figure 6:**
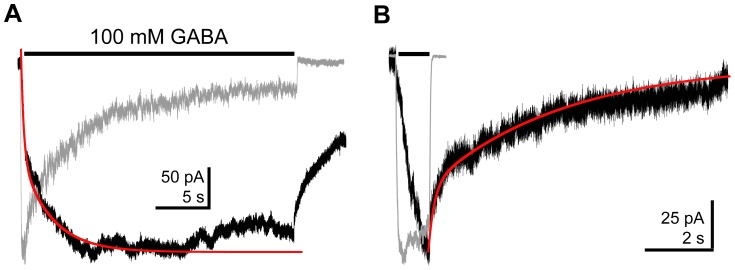
ELIC currents evoked by GABA exhibited unique kinetics. A) ELIC currents evoked by GABA (25 s, 100 mM) exhibited slow current activation, which was fit with a bi-exponential function (red curve). B) Following activation by a 1 s pulse of 100 mM GABA, ELIC current decayed very slowly and was fit with a bi-exponential function (red curve). Each GABA-evoked trace (black) is overlaid with a normalized response to 30 mM cysteamine obtained from a separate patch (gray) to highlight the differences in current activation and deactivation kinetics.

ELIC is also activated by the neurotransmitter GABA with an EC_50_ of 2.4 mM and maximal currents comparable to those elicited by cysteamine [7]. During applications of 100 mM GABA (the highest concentration we could achieve and still achieve the osmotic balance necessary to maintain a stable patch), ELIC currents activated much slower than cysteamine evoked currents, and had a τ_w_-activation from bi-exponential fits of 1.3±0.3 s, n = 3 (Figure 6A). The deactivation was also very slow following a 1 s GABA application with a τ_w_-deactivation of 4.6±1.2 s, n = 3 (Figure 6B).

### GLIC macroscopic kinetics

**Figure 7:**
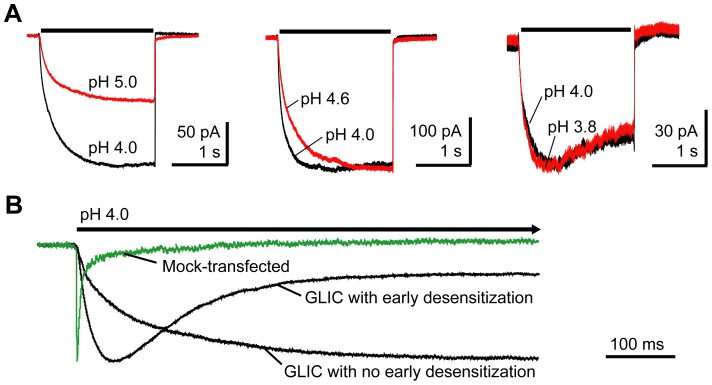
Proton induced GLIC currents. A) GLIC currents evoked by alternating 2 s jumps from pH 7.6 to the indicated values. Maximal current amplitudes were evoked by pH 4.0. B) A current recorded from an outside-out patch pulled from a mock-transfected HEK-293T cell evoked by pH 4.0 (green) is normalized and overlaid with GLIC currents (black) evoked by pH 4.0 from outside-out patches pulled from two different HEK-293T cells expressing GLIC to highlight differences in macroscopic current desensitization.

The proton-activated GLIC channel was also expressed in HEK-293T cells and studied using outside-out patch-clamp recording. We evoked GLIC currents by stepping from a solution buffered at pH 7.6 to a solution buffered at lower pH. Responses to pH 5.0, 4.6, 4.0, and 3.8 were compared (Figure 7A). The peak response reached a plateau at pH 4.0. HEK-293T cells endogenously express acid-sensing ion channels (ASICs) [19]. In patches from mock-transfected cells, stepping from pH 7.6 to pH 4.0 evoked small inward currents. However, these currents had small amplitudes (10–100 pA), ran down completely after two or three pulses, and displayed much faster activation and desensitization than currents observed in patches from GLIC transfected cells (τ-activation = 0.9±0.1 ms, n = 7; τ_w_-desensitization = 207±110 ms, n = 7; Figure 7B). The presence of ASIC or GLIC-mediated current was validated pharmacologically. Amiloride, a known blocker of ASICs [18], blocked currents evoked in patches from mock-transfected cells (Figure S1). Furthermore, picrotoxin had no effect on currents evoked in patches from mock-transfected cells, but blocked currents from GLIC-transfected cells (Figure S2). For all experiments, GLIC proton-mediated currents were measured after complete run down of endogenous ASIC current.

**Figure 8:**
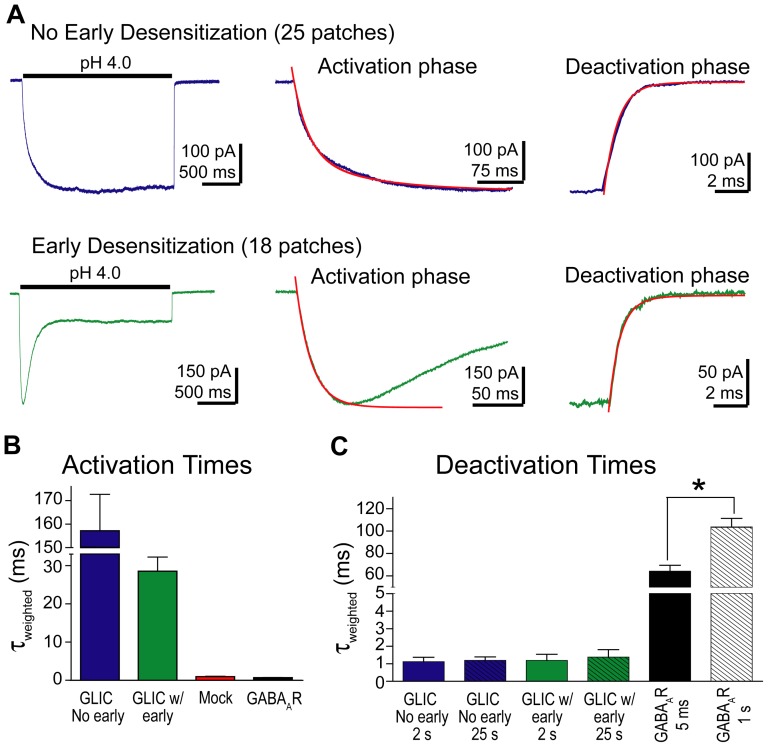
GLIC current activation was slow and deactivation fast compared to GABA_A_Rs. A) GLIC currents activated by a 2 s application of pH 4.0 buffer exhibited two different desensitization profiles. In 25 patches, little current desensitization was observed, whereas in 18 patches, a fast component of desensitization was observed. GLIC current activation and deactivation was analyzed separately for each group. Expanded views of current activation and deactivation are shown to the right and fit with bi-exponential functions (red curves). B) The τ_w_-activations for GLIC currents (pH 4.0) from both groups are plotted and compared to the τ_w_-activations of currents obtained from patches from mock-transfected cells (pH 4.0) and from GABA_A_Rs (10 mM GABA). Data are mean ± SEM (GLIC no early: n = 25, GLIC w/early: n = 18, Mock: n = 7). C) The τ_w_-deactivations are plotted for both groups of GLIC currents following a 2 s (as shown above) as well as a 25 s pulse of pH 4.0 buffer. The τ_w_-deactivations for GABA_A_R currents evoked by 5 ms or 1 s applications of 10 mM GABA are also plotted. Data are mean ± SEM (GLIC no early 2 s: n = 11, GLIC no early 25 s: n = 8, GLIC w/early 2 s: n = 6, GLIC w/early 25 s: n = 6).

Over the course of our experiments, we observed that GLIC proton-mediated currents (100–500 pA) exhibited one of two characteristic desensitization profiles. In 25 patches, little to no desensitization was observed during the first 2 seconds of pH 4.0 buffer application (Figure 8). In contrast, in 18 patches, a distinct fast desensitization was observed in the first 200 ms. Both desensitization profiles were observed on the same day of recording and from patches pulled from cells in the same dish. During repetitive pulses on a single patch, the desensitization profile always remained the same. Thus, we grouped our GLIC data into two categories: no early desensitization and early desensitization, and analyzed the kinetics of GLIC current activation, deactivation, and desensitization for each group separately.

GLIC current activation was measured at pH 4.0 and fit using a bi-exponential equation (Figure 8A). In patches with no early desensitization, the activation time course had a τ_w_-activation of 155±16 ms, n = 25; whereas currents with early desensitization had a τ_w_-activation of 28±4 ms, n = 18. For both groups, GLIC activation was significantly slower than GABA activation of the GABA_A_R (Figure 8B).

GLIC deactivation was measured following either a 2 s or 25 s pulse of pH 4.0 buffer. The time-course of deactivation was fit with a bi-exponential equation (Figure 8A). Irrespective of the length of pulse, the presence of early desensitization, or differences in the extent of desensitization, the τ_w_-deactivation was approximately 1 ms (No early desens. 2 s: 1.1±0.2 ms, n = 11; No early desens. 25 s: 1.2±0.2 ms, n = 8; Early desens, 2 s: 1.2±0.4 ms, n = 6; Early desens, 25 s: 1.4±0.4 ms, n = 6). These results are in contrast with GABA_A_R deactivation, which has a much slower time course and is dependent on the extent of desensitization (Figure 8C).

**Figure 9:**
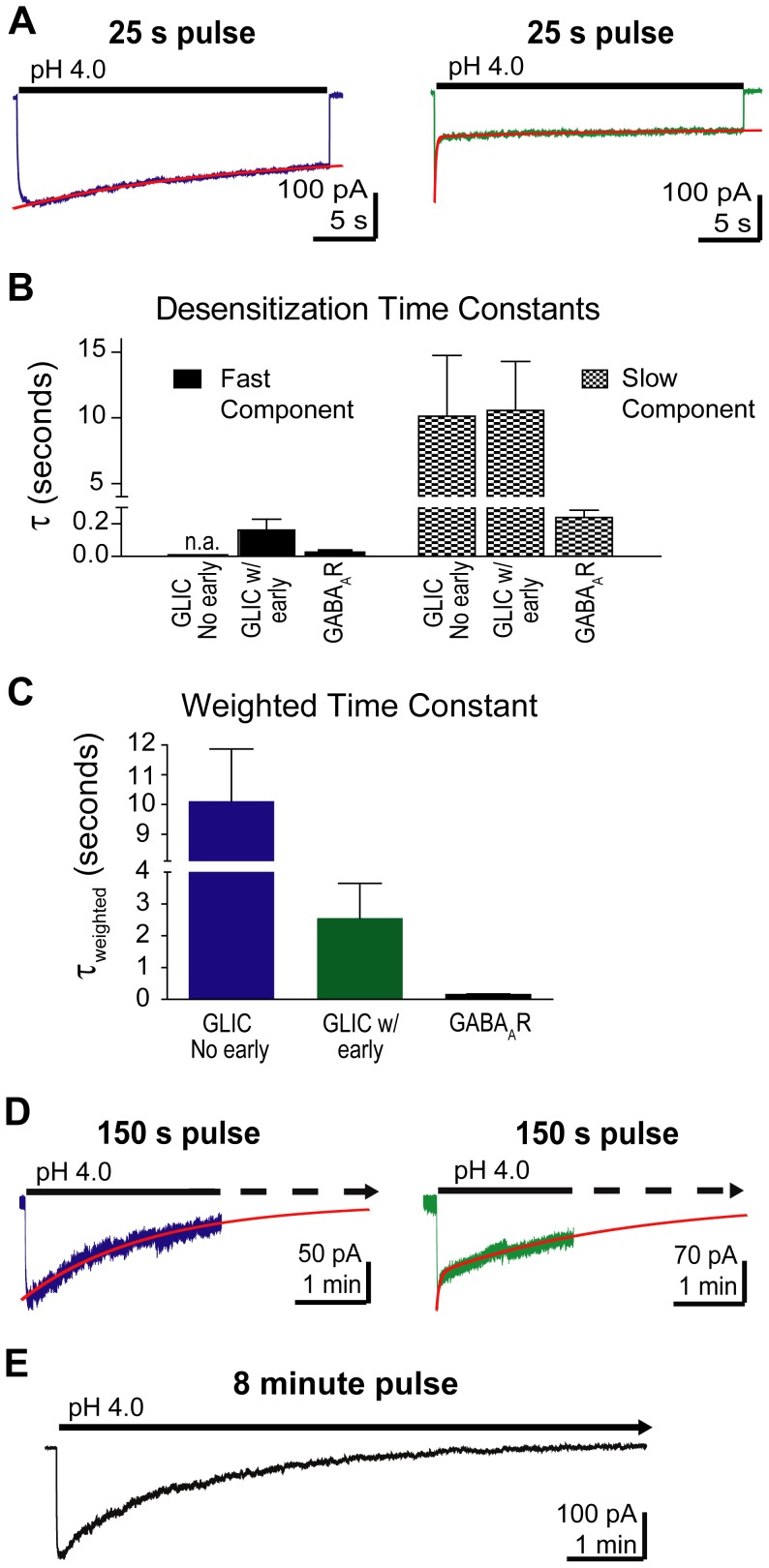
GLIC current desensitized slowly. A) GLIC current desensitization during a 25 s pulse of pH 4.0 buffer was fit with a single exponential when there was no early desensitization (left trace, red curve) or a bi-exponential function when early desensitization was present (right trace, red curve). B) Time constants for the fast and slow components of desensitization from both types of GLIC currents (pH 4.0, 25 s) and GABA_A_R currents (10 mM GABA, 1 s) are plotted. n.a. = not applicable, no measurable fast component. C) The τ_w_-desensitizations for both types of GLIC currents (pH 4.0, 25 s) and the GABA_A_R (10 mM GABA, 1 s) are plotted. Data are mean ± SEM (GLIC no early: n = 7, GLIC w/early: n = 9, GABA_A_R: n = 10). D) GLIC current desensitization during a 150 s pulse of pH 4.0 buffer was fit with a single exponential when there was no early desensitization (left trace, red curve) or a bi-exponential function when early desensitization was present (right trace, red curve). E) GLIC current decayed to baseline during an 8 minute pulse of pH 4.0 buffer.

In both groups of patches, current desensitization was observed during long 25 s pulses of pH 4.0 buffer (Figure 9A). Currents from patches with no early desensitization were fit with a single exponential and had a τ-desensitization of 10.1±1.7 s, n = 7, and 55±10% of the current remained at the end of 25 seconds. Currents from patches with early desensitization were fit with a bi-exponential function, yielding a fast τ-desensitization of 161±23 ms (59±8%) and a slow τ-desensitization of 10.6±1.2 s (13±4%), n = 9 (Figure 9B). At the end of the 25 s pulse, 28±5% of the current remained. The GLIC patches with fast desensitization still have a τ_w_-desensitization of 2.6±1.1 s, n = 9, which is significantly slower than the GABA_A_R (τ_w_-desensitization 162±37 ms, Figure 9C). We also measured desensitization during 2.5 minute long exposures to pH 4.0 buffer (Figure 9D). In these longer applications, currents from patches with no early desensitization were fit with a single exponential and had a τ-desensitization of about 1.8 min. Currents from patches with early desensitization were fit with a bi-exponential equation with a τ_fast_-desensitization of 2.6 s (22%) and a τ_slow_-desensitization of 2.7 min (78%). In the continued presence of pH 4.0 GLIC currents fully desensitized after 8 minutes (Figure 9E).

## Discussion

Here, we describe the macroscopic current kinetics of two prokaryotic pLGICs, ELIC and GLIC, and compare them to the GABA_A_R, a eukaryotic channel. We demonstrate that the prokaryotic channels have very slow activation, slow desensitization, and fast deactivation compared to the GABA_A_R.

Previous reports of GLIC activation found a rise time of 260 ms using whole cell recordings of HEK cells with a pH jump to 5.0 [2], 19 ms using outside-out HEK patches with a pH jump to 4.5 [13], and 11 ms using purified GLIC channels reconstituted in proteoliposomes with a pH jump to 2.5 [15]. Here, in GLIC patches with no early desensitization, we measured a τ_w_-activation of 155 ms similar to Bocquet et al. (2007) [2], who also did not observe any substantial current desensitization. When we did observe desensitization, our τ_w_-activation was faster (28 ms) and is similar to that reported by Gonzalez-Gutierrez et al. (2012) [13], who also observed GLIC current desensitization. The fastest GLIC activation time, which was measured by Velisetty and Chakrapani (2012) [15], was obtained from detergent purified GLIC protein reconstituted in asolectin membranes. Lipid composition can significantly impact channel kinetics [15], [20], which may partially explain the differences in GLIC kinetics reported.

Our measured activation of ELIC currents evoked by cysteamine (48±4 ms) was similar to the two previous reports examining ELIC kinetics (20±5 ms and 84.6±7 ms), both of which used excised patches from oocytes [7], [13]. ELIC activation was faster than GLIC, but currents from both prokaryotic channels activated significantly slower than GABA_A_R. Surprisingly, GABA-evoked currents from ELIC activated very slowly (∼1 s) and deactivated even slower (∼5 s). While not specifically highlighted, the slow kinetics of GABA-evoked currents has been seen in other studies on ELIC [7], [9]. Why the kinetics of GABA-evoked currents from ELIC are so much slower than cysteamine-evoked currents requires further investigation but suggests GABA is not a typical agonist. The slow current onset and offset is reminiscent of the kinetics of neurosteroid direct activation of the GABA_A_R, which has been attributed to slow partitioning of the neurosteroid into the lipid bilayer and slow wash-out [21].

The significantly slower activation of prokaryotic channels compared to GABA_A_Rs and other eukaryotic channels is emerging as a general feature that distinguishes these evolutionarily distant homologues. Most eukaryotic pLGICs exhibit sub-millisecond activation times, and a review of literature on eukaryotic pLGICs reveals even the slowest isoforms have rise times in the millisecond range (3 ms for α3β4 nACh receptors [13] and 2.5 ms to 7.3 ms for 5-HT3A receptors [22], [23]. The slow prokaryotic activation times suggest that structural elements important for mediating their activation differ from eukaryotic channels. Previous work has shown that loose packing of the β-sandwich hydrophobic core in the extracellular binding domain of eukaryotic pLGICs is a unique structural feature that is absent in the prokaryotic channels, and contributes to their ability to rapidly switch from closed to open channel states when agonists are bound [24]. Additionally, an interaction between the eponymous cys-loop and the M2-M3 loop in eukaryotic pLGICs has been implicated in the control of fast gating [25]. The sequences of these elements are poorly conserved between eukaryotic and prokaryotic channels suggesting that the interaction may not be optimal in prokaryotic channels, leaving the extracellular domain and the ion channel gate poorly coupled.

Determining whether ligand-bound channels undergo a transition to closed desensitized states is important for understanding what functional state the channel might be in when interpreting data from steady state structural experiments such as X-ray crystallography and electron paramagnetic resonance spectroscopy. An early study on GLIC, using HEK whole-cell recordings, reported little current desensitization during a 30 s application of pH 5.0 buffer [2]. In oocytes, using two-electrode voltage clamp, Parikh et al. (2011) observed very slow current desensitization with a time constant of 28 s at pH 4.0 [14], whereas in HEK outside-out patches at pH 4.5, Gonzalez-Gutierrez and Grosman (2010) reported a GLIC desensitization time constant of 1.4 s [26]. More recently, using patch recordings from GLIC reconstituted in liposomes, Velisetty and Chakrapani (2012) reported a GLIC desensitization time constant of 1.6 s at pH 2.0 [15].

Here, in HEK outside-out patches at pH 4.0, we also show that GLIC currents desensitize but with two distinct kinetic profiles. In 25 out of 43 patches, currents desensitized with slow kinetics, whereas in 18 out of 43 patches, a fast and slow component of desensitization was detected. This variability in GLIC desensitization kinetics requires further investigation, but several possibilities exist. Our HEK-293T cells are presumed to be homogenous but different cell types may exist that contain different endogenous proteins that alter GLIC desensitization kinetics differently. Also, the expression level of GLIC may vary from cell to cell. Patches containing GLIC clusters may have different kinetics than patches containing channels that are evenly dispersed [27]. Additionally, the membrane composition may vary from patch to patch, which could alter channel kinetics. For example, increasing the cholesterol content of the membrane increased GLIC desensitization [15]. A similar phenomenon was observed in α7 nAChRs, where a reduction in cholesterol and sphingomyelin was shown to reduce the amount of fast desensitization [20].

Kinetic models of the eukaryotic GABA_A_R take into account the observed coupling of desensitization and deactivation. This coupling is known to influence the time course of inhibitory post-synaptic currents [18], [28]. The time required for deactivation after removal of agonist increases in proportion to the extent of desensitization [18]. After agonist removal, the current decay is governed by a variety of transitions including desensitization and resensitization transitions, opening and closing transitions, and agonist unbinding steps. Upon agonist removal, receptors can flow out of desensitized states and re-open before agonist dissociation. This prolongs the deactivation phase. In ELIC and GLIC, when desensitization was observed there was no such coupling. Not every cys-loop receptor displays desensitization-deactivation coupling, for example the deactivation time course of the α1 glycine receptor is independent of the length of agonist application and extent of desensitization [29]. Structurally, transmembrane segments 1 and 2 appear to be involved as mutations of residues in these segments remove the coupling between desensitization and deactivation [30], [31].

With the growing use of ELIC and GLIC as models for investigating the mechanisms underlying eukaryotic pLGIC gating and allosteric drug modulation, it is important to establish how well their functional properties resemble the properties of eukaryotic channels. Here, we show ELIC and GLIC activate and desensitize in a similar fashion as eukaryotic pLGICs but with slower kinetics. Our detailed characterization of the macroscopic kinetics of ELIC and GLIC will aid in correlating functional conformation states with emerging crystal structures. The functional variations between prokaryotic and eukaryotic pentameric channels described here are likely due to differences in specific structural elements, the identification of which will help reveal molecular mechanisms involved in pLGIC gating.

## Supporting Information

Figure S1 **Endogenous proton-activated currents are blocked by amiloride.** Outside-out patch-clamp recordings from mock-transfected HEK-293T cells. Currents were evoked by alternating 2 s jumps from pH 7.6 to pH 6.0 or pH 6.0 containing 25 µM amiloride. The jumps were interleaved by 30 s washes. The traces are the ensemble averages of 5 repeated pairs of applications.
(TIF)

Figure S2 **Picrotoxin selectively blocks GLIC currents.** Outside-out patch-clamp recordings performed with HEK-293T cells examined the effect of 50 µM picrotoxin (ptx) on proton-activated currents. A) Currents evoked by alternating jumps from pH 7.6 to pH 6.0 in an outside-out patch from a mock-transfected cell were unaffected by picrotoxin. Jumps to pH 6.0 were used instead of pH 4.0 because pH 6.0 evoked stable currents during repeated applications. B) Currents evoked by alternating jumps from pH 7.6 to pH 4.0 in an outside-out patch from a GLIC-transfected cell were blocked by picrotoxin. The ensemble averages of several repeated pairs of applications are overlaid in A and B.
(TIF)
