# Supplementary figures and images for: Macroscopic Kinetics of Pentameric Ligand Gated Ion Channels: Comparisons between Two Prokaryotic Channels and One Eukaryotic Channel
Source: PLoS One. 2013 Nov 19;8(11):e80322. doi: 10.1371/journal.pone.0080322 (PMC3833957; doi:10.1371/journal.pone.0080322)

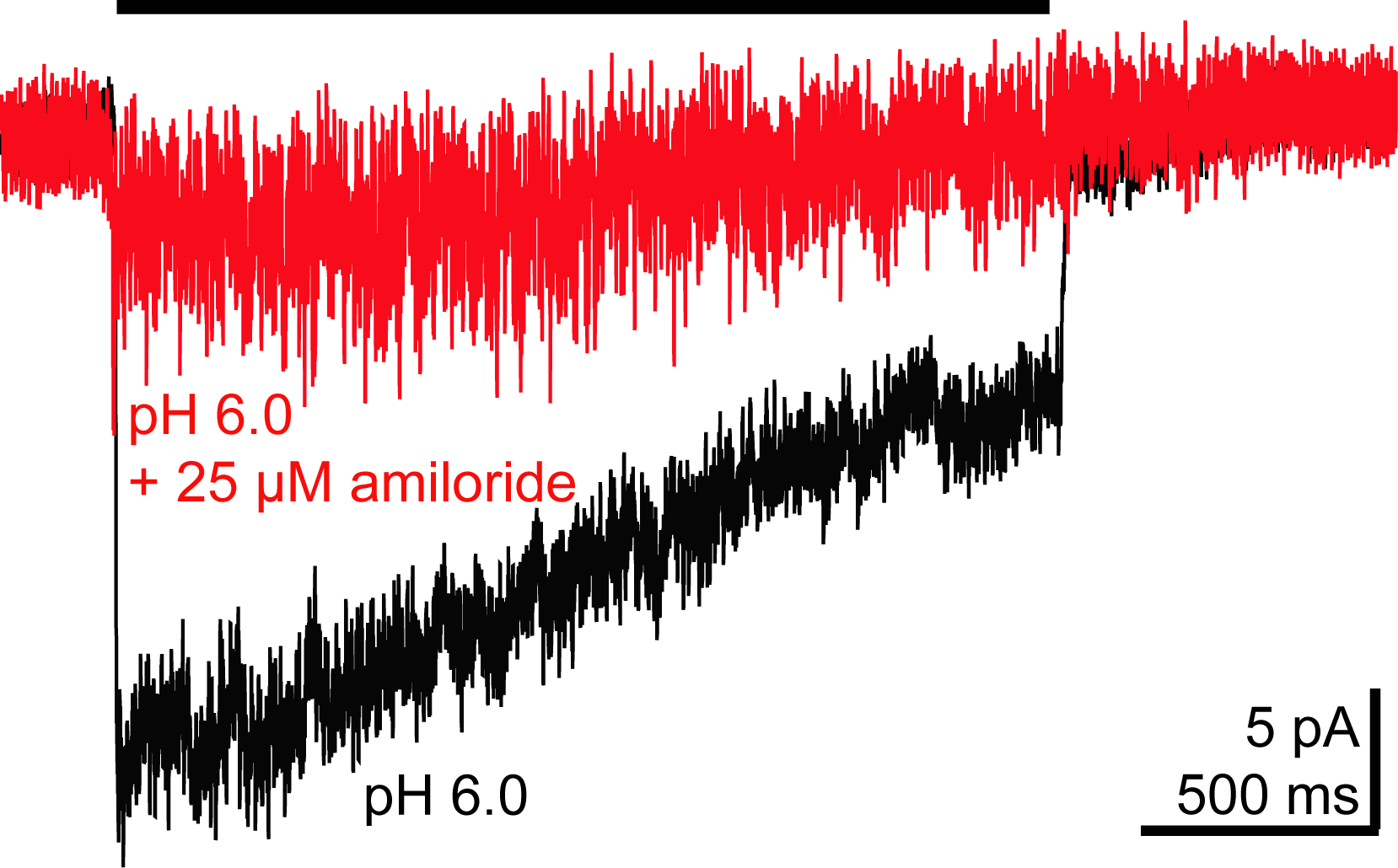

Supplement: Figure S1 — Endogenous proton-activated currents are blocked by amiloride. Outside-out patch-clamp recordings from mock-transfected HEK-293T cells. Currents were evoked by alternating 2 s jumps from pH 7.6 to pH 6.0 or pH 6.0 containing 25 µM amiloride. The jumps were interleaved by 30 s washes. The traces are the ensemble averages of 5 repeated pairs of applications. (TIF) [file pone.0080322.s001.tif]

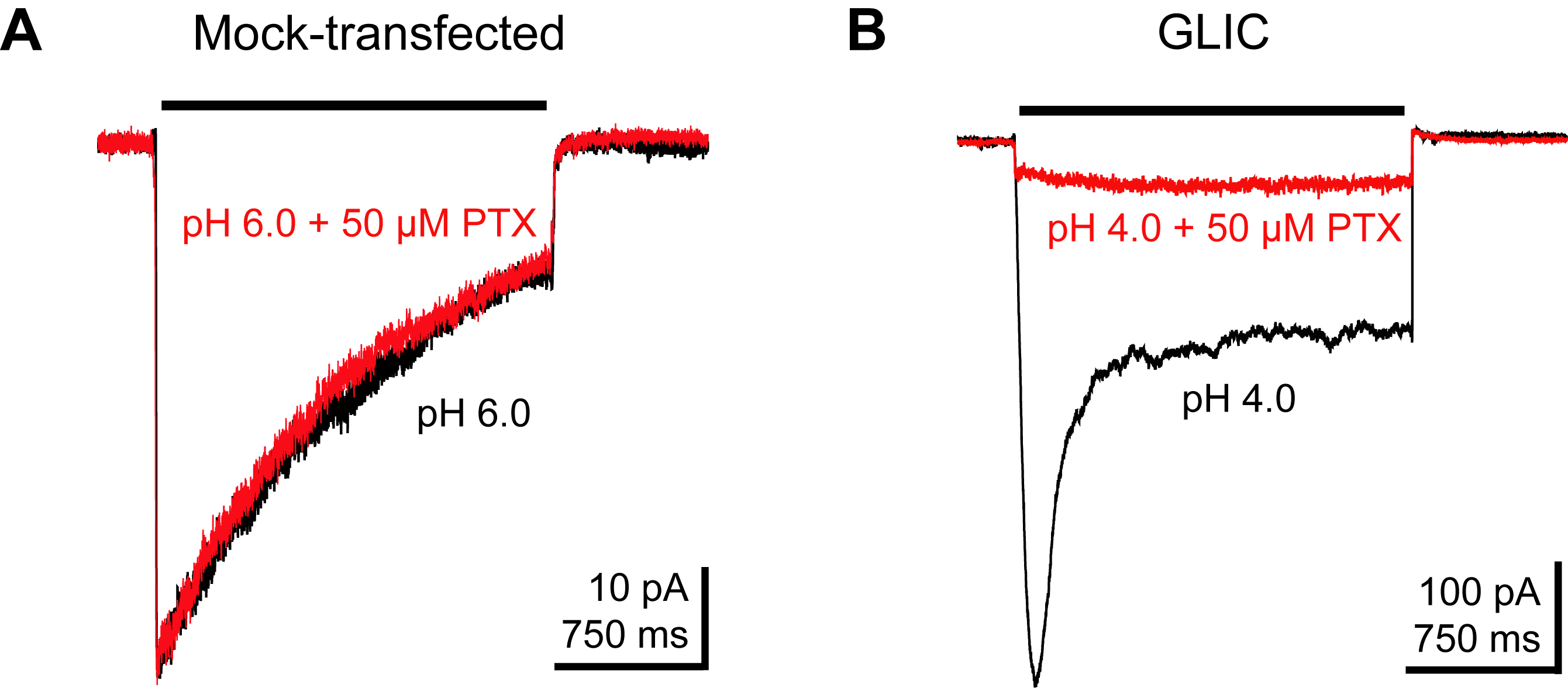

Supplement: Figure S2 — Picrotoxin selectively blocks GLIC currents. Outside-out patch-clamp recordings performed with HEK-293T cells examined the effect of 50 µM picrotoxin (ptx) on proton-activated currents. A) Currents evoked by alternating jumps from pH 7.6 to pH 6.0 in an outside-out patch from a mock-transfected cell were unaffected by picrotoxin. Jumps to pH 6.0 were used instead of pH 4.0 because pH 6.0 evoked stable currents during repeated applications. B) Currents evoked by alternating jumps from pH 7.6 to pH 4.0 in an outside-out patch from a GLIC-transfected cell were blocked by picrotoxin. The ensemble averages of several repeated pairs of applications are overlaid in A and B. (TIF) [file pone.0080322.s002.tif]
